# Topical citicoline and vitamin B12 versus placebo in the treatment of diabetes-related corneal nerve damage: a randomized double-blind controlled trial

**DOI:** 10.1186/s12886-020-01584-w

**Published:** 2020-08-01

**Authors:** Paolo Fogagnolo, Ettore Melardi, Laura Tranchina, Luca Rossetti

**Affiliations:** 1 Eye Clinic, Università degli Studi di Milano; ASST Santi Paolo e Carlo, San Paolo Hospital, Via di Rudini’, 8, 20142 Milan, Italy; 2 ASST Rhodense, Garbagnate Milanese Hospital, Milan, Italy

**Keywords:** Diabetes, Cornea innervation, Corneal sensitivity, Citicoline, Vitamin B12, Dry eye disease

## Abstract

**Background:**

To evaluate the effects of topical citicoline and vitamin B12 (Cit-B12: OMK2, Omikron Italia srl, Italy) on corneal innervation of patients with diabetic neuropathy.

**Methods:**

This prospective, randomized, double blind, placebo-controlled study included 30 patients randomised with a 2:1 ratio to Cit-B12 or placebo 3 times daily for 18 months. At baseline and at months 4, 8, 12, 18 patients underwent the Ocular Surface Disease Index questionnaire (OSDI), tear break-up time, evaluation of corneal and conjunctival staining, Schirmer I test, Cochet-Bonnet esthesiometry, and confocal biomicroscopy of corneal sub-basal plexus (SBP). Fiber lenght density (FLD) was calculated using NeuronJ and expressed in mm/mm2. Raw data and differences from baseline were analysed in the two groups.

**Results:**

29/30 patients concluded the study. The two groups had similar FLD at baseline; it progressively improved up to month 18 in both groups (Cit-B12, *p* < 0.0001; controls, < 0.0001–0.03); improvement at month 18 vs baseline was higher in Cit-B12 than placebo (33% vs 15%, *p* = 0.04). A progressive amelioration of corneal sensitivity (baseline, 28 ± 18 mm; month 18, 52 ± 10 mm, *p* < 0.0001), conjunctival staining (*P* = 0.04) and OSDI questionnaire (*P* = 0.05) were shown on Cit-B12 group alone. Both treatments were well tolerated and adherence during the study was high.

**Conclusions:**

Cit-B12 ameliorated both morphology and function of corneal nerves in patients with diabetes, thus suggesting a neuroregenerative effect.

**Trial registration:**

Trial registration NCT03906513, retrospectively registered on 08 April 2019.

## Background

Cornea innervation plays a key role in the homeostasis of the cornea and the ocular surface. Nerve modifications of the cornea subbasal nerve plexus (SBP) occur in several conditions such as keratoconus, infectious keratitis, corneal dystrophies, neurotrophic keratopathy, long-standing contact lens wear, eye surgeries, and diabetes [1]. SBP can be clinically evaluated using confocal microscopy, a non-invasive technique whose clinical relevance is well-recognized in the diagnosis of ocular surface pathologies [2].

In patients with diabetes, SBP loss correlates with the severity of systemic neuropathy [3] and, more generally, with the severity of diabetes [4]; also, SBP density improves after pancreas transplantation [4, 5]. These findings suggest that corneal confocal microscopy may be a valid tool for monitoring diabetic neuropathy.

Recently, eyedrops and surgical strategies that specifically target axonal regeneration of corneal nerves have started to become available; these include nerve growth factor [6, 7], coenzyme Q10 [8], and corneal neurotization [9, 10]. Among possible treatments, citicoline (associated with vitamin B12) facilitates the recovery of cell membrane damage. Several experimental studies in vitro and in vivo have suggested the neuroprotective effects of citicoline and vitamin B12. Systemic administration of citicoline has been found to counteract neuronal cell damage in animal models of cerebral ischemia [11]; vitamin B12 supplementation ameliorated the peripheral nerve lesions in experimental diabetic neuropathy [12, 13]. In human studies, recent data suggest that administration of systemic citicoline may slow down neurodegenerative diseases such as glaucoma [14]. Topical administration of citicoline was effective in reducing neurodegeneration in models of diabetic and non-diabetic retinal degeneration, both in-vitro [15] and in-vivo [16, 17]. Topical citicoline also proved to be a viable neuroprotective treatment in glaucoma patients [18, 19] and, very recently, also on the inner retina of patients with diabetes [20]. Currently there are no clinical data on the effects of the treatment with both citicoline and vitamin B12 eye drops on corneal nerves.

Aim of this study was to test the hypothesis that an eyedrop containing citicoline and vitamin B12 may stimulate SBP axonal regrowth in patients with diabetes.

## Methods

The study was a single-centre, randomized, double-blind, placebo-controlled, prospective study on 30 patients with diabetes. It was conducted at the Eye Clinic of San Paolo Hospital, Università degli Studi, Milan, Italy and conducted according to the tenets of the Declaration of Helsinky. It was funded by Omikron Italia s.r.l., Rome, Italy and registered (clinicaltrials.gov, ID NCT03906513, retrospectively registered on 08 April 2019); it adhered to CONSORT guidelines. Informed consent was obtained from all patients prior to enrollment.

Inclusion criteria were: age > 18 years and patients with type 1 or type 2 diabetes who received Argon Laser Photocoagulation. Exclusion criteria were: neuropathy of any other cause than diabetes; history of conditions known to affect corneal sensitivity; coexisting other corneal diseases; autoimmune diseases; Sjogren syndrome; history of corneal trauma; contact lenses use; patients needing eye surgery or who received eye surgery at least 180 days before inclusion; contraindications to the use of any active substances and/or excipients; pregnant and lactating women.

Patients were randomized (list of random number) with a 2:1 ratio to two treatment arms: 20 patients were treated with active treatment Cit-B12 (OMK2, Omikron Italia srl, Italy: 2% citicoline, 0.2% hyaluronic acid with molecular weight of 700–900 KiloDalton, 0.02% cyanocobalamin, and 0.01% benzalkonium chloride) and 10 with placebo (0.3% hypromellose, SoftDrops, Farmigea S.p.A., Italy) given three times daily (8 am, 2 pm, 8 pm) for the duration of the study. Weighted randomization was used in order to balance in the two groups the following characteristics, known to affect SBP: duration of the disease, number of cases of insulin-dependent diabetes, and baseline SBP density.

The study consisted on 5 visits: baseline, month 4, month 8, month 12, month 18.

At each visit, the following examinations were performed in the following order: questionnaire of symptoms using the Ocular Surface Disease Index (OSDI, Allergan, Irvine, USA); anterior segment ophthalmoscopy; tear film fluorescein break-up time (TBUT), defined as the number of seconds that elapse between the last blink and the appearing of the first dry spot in tear film (TBUT was the mean of 2 consecutive measures); grading of corneal staining with fluorescein using Oxford scale; grading of conjunctival staining with fluorescein using Van Bijelsterveld scale; Schirmer I test; measure of central corneal sensitivity by Cochet-Bonnet esthesiometer; confocal biomicroscopy of the central SBP of the cornea. This examination was performed using in-vivo confocal microscopy (HRT II Cornea Module, Heidelberg Engineering GmbH, Heidelberg, Germany); at each visit, 9 central images not overlapping by more than 20% were selected according to the strategy suggested by Vagenas et al. [21].

Blinding between operators was strictly maintained during the study. Three operators per patient were involved: confocal operator, clinical operator, and adherence operator (assessing at each visit adherence to treatment, stability of diabetes and presence of side effects by inspecting patients’ diary). The study was designed to exclude patients who missed more than 3 consecutive days of treatment (a fact which did not occur).

### Statistical analysis

The primary outcomes of the study were the change in fiber length density (FLD) of SBP and sensitivity occurring at each visit vs baseline. The secondary outcomes were the changes in clinical signs (TBUT, Schirmer I, corneal and conjunctival epithelial staining) and symptoms (OSDI) of ocular surface damage.

Sample size was calculated using previous data on nerve density at confocal microscopy (which was the main outcome of the study). There is high span of literature data on corneal nerve density in diabetes, due to differences on confocal microscope, heterogeneity of diabetic populations, differences on data collection; there is also paucity of data on the efficacy of treatments to improve corneal axonal growth. We used two studies showing increase of SBP density after pancreas cell transplantation [4, 5]; we assumed a mean baseline value of 11.8 no/mm^2^ and a 18-month value of 14.2 no/mm^2^. Using alpha = 0.05, beta = 0.9, a worth-detecting difference of 20% from baseline and a two tailed test, about 15 patients on active treatment were necessary; assuming a 20% drop-out per year, 20 patients were enrolled. The placebo group was not relevant for sample size calculation and therefore was not the object of sample size calculation; 10 patients were enrolled.

Previous to the study, we tested test-retest variability of confocal microscopy on five patients for each operator in two consecutive sessions and also both inter- and intra-evaluators tracing agreement on five patients. Intra- and inter-class correlation coefficients, ICC, were calculated.

Study analysis was performed on the worse eye of each patient (the one with lower SBP density). The images to be used for the analyses were selected and anonymized by the confocal operator at the end of each visit. All images were analyzed at the end of the study, with the same operator analyzing all the images of a given patients. For the 9 images collected at each visit, axons were manually traced and axon length per field was calculated using NeuronJ software by ImageJ (http://imagej.nih.gov/ij/; provided in the public domain by the National Institutes of Health, Bethesda, MD, USA). FLD was defined as the mean of the 9 confocal fields and expressed in mm/mm2 [21].

OSDI questionnaire was classified as normal (values ranging between 0 and 12%), mild (13–22%), moderate (23–32%).

Missing data (7 patients missed one visit each) were managed using the mean value of the previous visit(s) for the given patient. Dataset for primary and secondary outcomes were analysed by means of analysis of covariance, using treatment as covariate. Raw data and percentage change from baseline were studied. In the case of positive results, inter-visit differences were inspected by means of t-test and X2 (respectively for continuous and categorical variables). Correlation between FLD and esthesiometry were calculated using Pearson r.

## Results

29/30 patients concluded the study; one patient withdrawn from the trial after month 4 for personal reasons; his data were not considered in the analysis. No differences were shown for age (66 ± 8 years in Cit-B12 vs 64 ± 10 in placebo, *P* = 0.87), sex (60% females in both groups), ethnicity (90% Caucasian in both groups). Duration of the disease was 7.8 ± 4.4 in Cit-B12 and 6.5 ± 6.2 years in placebo (*P* = 0.24); the percentage of insulin-dependent diabetes was 30% in both groups; no changes in diabetic treatment occurred during the study and all patients were stable in the course of the study according to routine follow-up diabetologic examinations.

Test-retest intraoperator agreement at confocal microscopy was good (ICC of 0.63, 0.73, 0.71) and both inter- and intra-operator agreement with ImageJ were excellent (respectively > 0.83; and 0.90, 0.90, 0.94).

Analysis of variance was statistically significant for change in SBP vs baseline (*P* = 0.012), esthesiometry (*P* = 0.003), change in esthesiometry vs baseline (*P* = 0.01); a borderline *p*-value of 0.078 was obtained for SBP density.

**Fig. 1:**
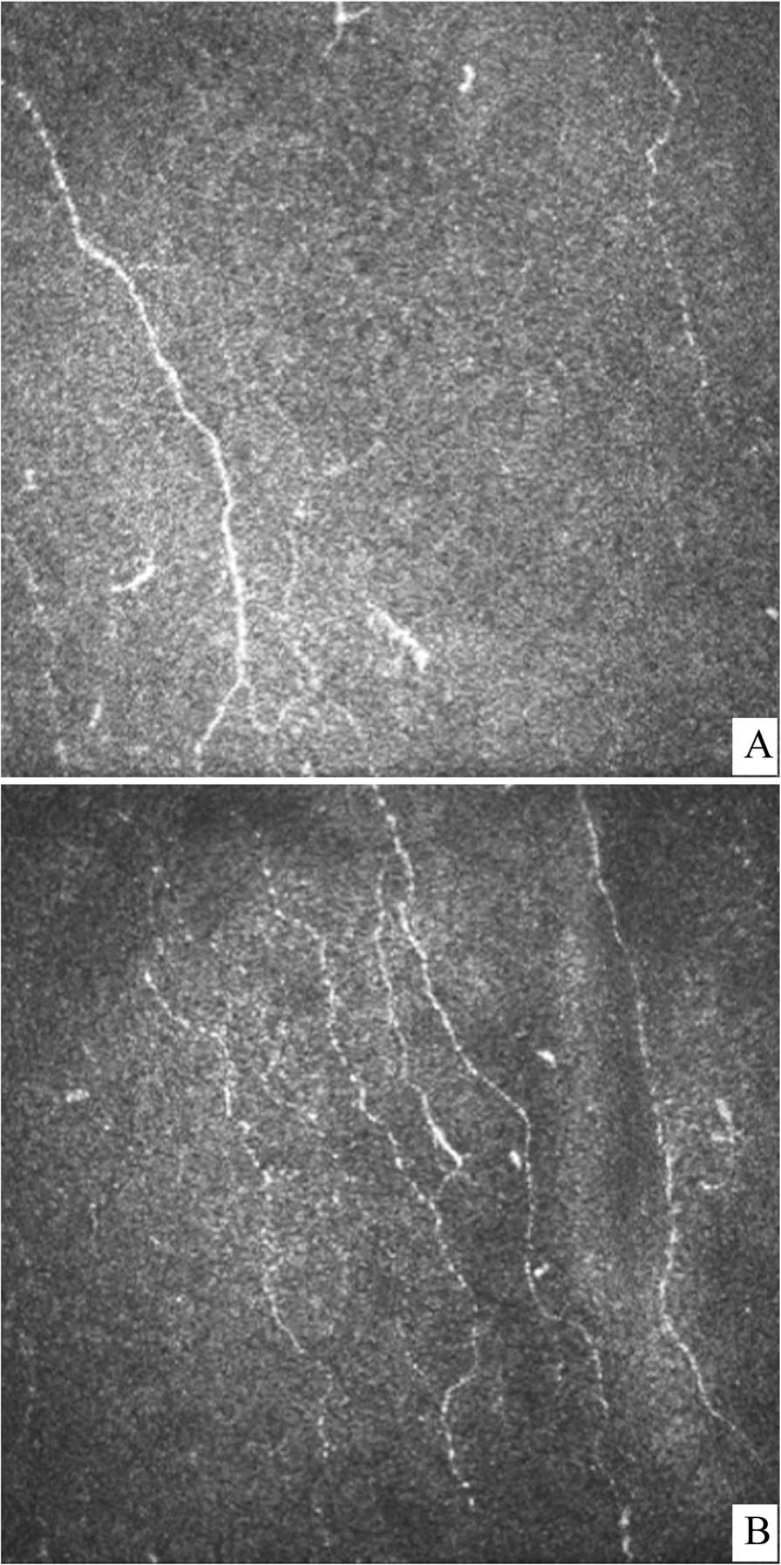
An example of the effects of Cit-B12 during the study. Confocal images of the central cornea at a) baseline (FLD 7.1 mm/m2) and b) 18 months (FLD 10.3 mm/mm2)

Findings at confocal microscopy and corneal sensitivity were then compared in the two groups. Tables 1 and 2 respectively show SBP density in the two groups and percentage of change. The two groups had similar SBP density at baseline; both groups showed a progressive amelioration of data compared with baseline (*p* < 0.0001 for Cit-B12, P ranging from < 0.0001 and 0.03 for placebo group), and between month 4 and month 8 (*p* = 0.03 for both Cit-B12 and placebo group). Considering percentage change, at the end of follow-up Cit-B12 showed a statistically significant improvement of corneal SBP compared with placebo: 33% vs 15% (*p* = 0.04; Fig. 1).

**Table 1:** Corneal FLD (mm/mm2)

	Cit-B12	Placebo	*P*
Baseline	10.6 ± 2.9	12.7 ± 2.9	0.09
Month 4	13.4 ± 4.1*	15.8 ± 3.9*	0.07
Month 8	14.3 ± 4.1*	16.5 ± 2.9 *	0.08
Month 12	12.8 ± 4.1*	14.8 ± 3.0§	0.10
Month 18	14.2 ± 4.6*	14.4 ± 4.0^	0.43

*, *p* < 0.0001; §, *p* = 0.01, ^, *p* = 0.03. All comparisons are vs baseline

**Table 2:** Corneal FLD: percentage improvement vs baseline

	Cit-B12	Placebo	*P*
Month 4	27% ± 23% (+ 63%; − 14%)	26% ± 18% (+ 49%; − 2%)	0.43
Month 8	37% ± 27% (+ 94%; − 9%)	34% ± 30% (+ 98%; − 2%)	0.39
Month 12	21% ± 29% (+ 66%; − 44%)	19% ± 17% (+ 50%; − 4%)	0.40
Month 18	33% ± 26% (+ 88%; − 9%)	15% ± 25% (+ 42%; − 39%)	**0.04**

Tables 3 and 4 respectively show the results of esthesiometry and percentage change. Cit-B12 showed a progressive amelioration of corneal sensitivity, which was statistically significant at each visit compared with the previous one (month 4 vs baseline, *p* < 0.0001; month 8 vs month 4, *p* = 0.002; month 12 vs month 18, *p* = 0.03, month 18 vs month 12, *p* = 0.05; all visits compared with baseline, *p* < 0.0001). In placebo group, the change of sensitivity was not statistically significant comparing any timepoint (*p* > 0.06). Considering percentage change, Cit-B12 obtained better improvements than placebo at each timepoint (*p* < 0.01). The correlation between FLD improvement and corneal sensation improvement was not statisticallly significant (r = 0.27, *P* = 0.24).

**Table 3:** Corneal esthesiometry (mm)

	Cit-B12	Placebo	*P*
Baseline	28 ± 18	41 ± 16	0.07
Month 4	40 ± 16*	42 ± 16	0.79
Month 8	49 ± 10*	50 ± 11	0.85
Month 12	50 ± 12*	48 ± 12	0.70
Month 18	52 ± 10*	50 ± 13	0.63

*, *p* < 0.0001 vs baseline

**Table 4:** Corneal esthesiometry: percentage improvement vs baseline

	Cit-B12	Placebo	*P*
Month 4	73% ± 79% (+ 317%; − 17%)	5% ± 11% (+ 27%; − 14%)	**0.001**
Month 8	143% ± 142% (+ 533%; − 31%)	44% ± 76% (+ 219%; − 18%)	**0.02**
Month 12	135% ± 115% (+ 338%; − 24%)	39% ± 82% (+ 244%; − 27%)	**0.01**
Month 18	153% ± 121% (+ 362%; − 27%)	44% ± 78% (+ 231%; − 37%)	**0.007**

**Fig. 2:**
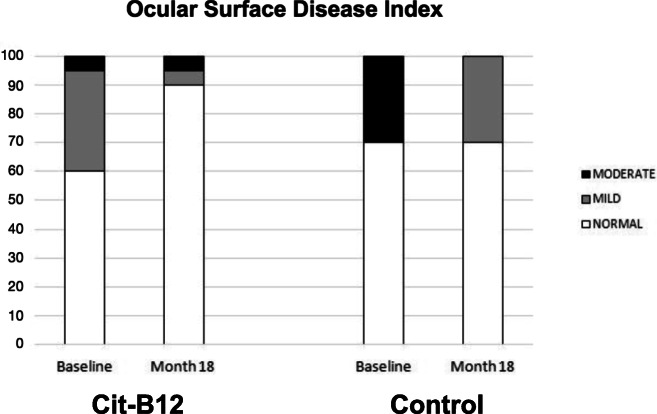
Changes of the frequency of stages of the Ocular Surface Disease Index at baseline vs month 18 in the two groups (Cit-B12, *P* = 0.05). Normal, OSDI = 12 or less; mild, OSDI between 13 and 22; moderate, OSDI between 23 and 32

**Fig. 3:**
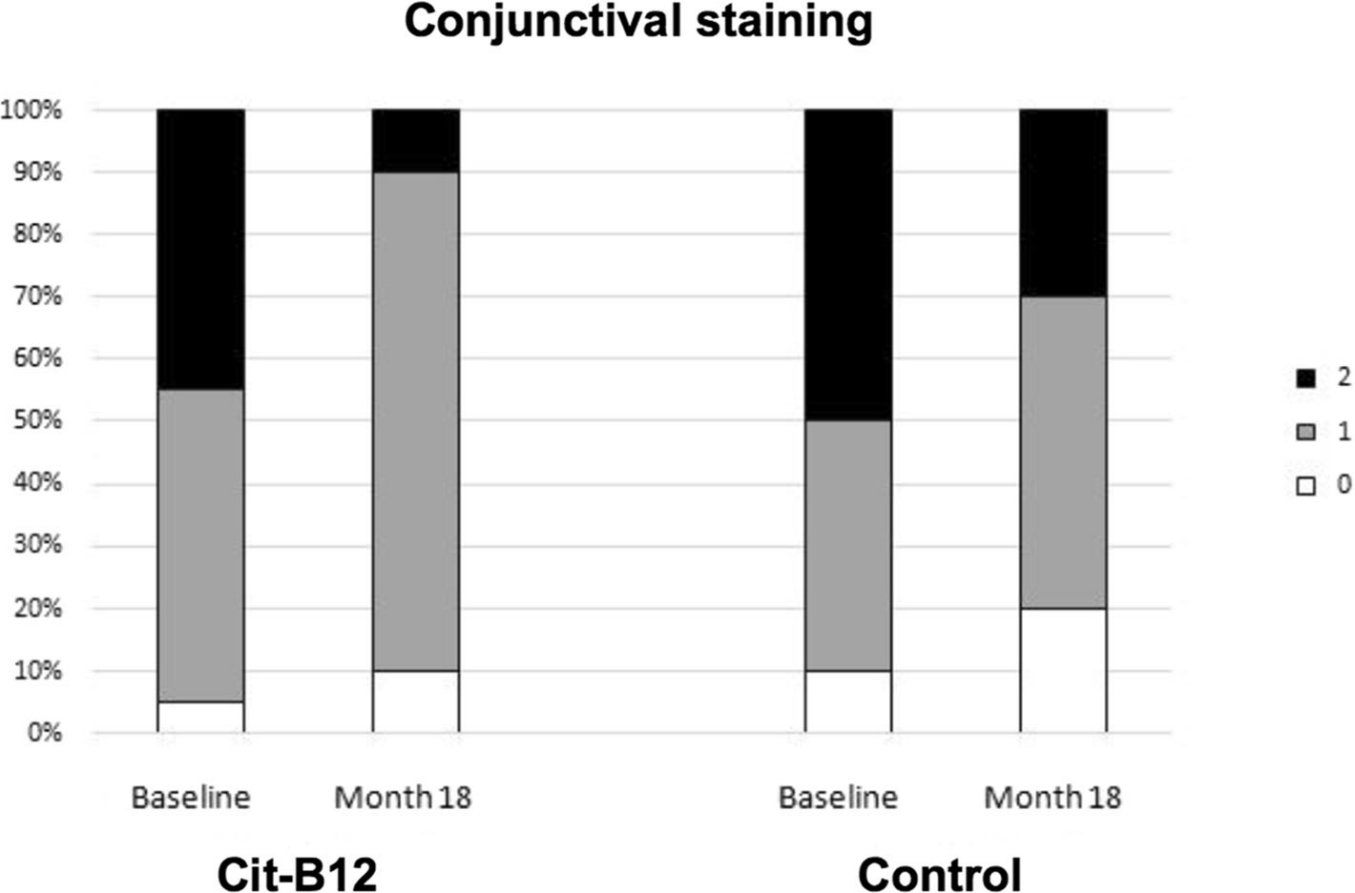
Changes of conjunctival staining according to Van Bijelsterveld scale at baseline vs month 18 in the two groups (Cit-B12, *P* = 0.04)

Table 5 shows data of ocular surface signs and symptoms in the two groups at each visit. No statistically significant changes occurred during the study, apart from the reduction of OSDI and conjunctival staining occurring on Cit-B12 group at month 18 vs baseline (Figs. 2 and 3).

**Table 5:** Comparison of ocular surface parameters in the two groups at each timepoint of the study

**BUT**	baseline	4 m	8 m	12 m	18 m
Cit-B12	10.1 ± 5.7	9.0 ± 5.9	9.7 ± 7.2	9.6 ± 4.7	9.8 ± 6.3
Placebo	8.6 ± 5.5	7.8 ± 5.0	7.4 ± 6.3	10.9 ± 6.5	9.4 ± 5.5
p	0.48	0.56	0.39	0.62	0.87
**SCHIRMER**	baseline	4 m	8 m	12 m	18 m
Cit-B12	15.9 ± 8.9	14.8 ± 7.1	14.7 ± 7.7	16.5 ± 9.0	14.4 ± 9.2
Placebo	14.1 ± 8.8	14.8 ± 9.2	15.5 ± 7.6	17.5 ± 11.1	16.4 ± 11.0
p	0.59	0.99	0.80	0.83	0.66
**CONJUNCTIVAL STAINING**					
	baseline	4 m	8 m	12 m	18 m
Cit-B12	1.40 ± 0.60	1.60 ± 0.50	1.21 ± 0.42	1.11 ± 0.32	1.00 ± 0.47
Placebo	1.40 ± 0.70	1.40 ± 0.70	1.10 ± 0.57	1.13 ± 0.35	1.25 ± 0.46
p	0.99	0.43	0.60	0.89	0.22
**CORNEAL STAINING**					
	baseline	4 m	8 m	12 m	18 m
Cit-B12	0.20 ± 0.41	0.40 ± 0.50	0.21 ± 0.42	0.05 ± 0.23	0.11 ± 0.32
Placebo	0.30 ± 0.48	0.20 ± 0.42	0.10 ± 0.32	0.01 ± 0.00	0.13 ± 0.35
p	0.58	0.26	0.43	0.33	0.89
**OSDI**	baseline	4 m	8 m	12 m	18 m
Cit-B12	9 ± 7	8 ± 7	7 ± 7	6 ± 5	8 ± 10
Placebo	13 ± 11	8 ± 6	7 ± 5	9 ± 7	8 ± 8
p	0.30	0.90	0.90	0.21	0.98

According to OSDI classification, the prevalence of dry eye was 36% at the beginning of the study (40% in Cit-B12 group, 7 mild, 1 moderate; 30% in placebo group: 0 mild, 3 moderate), and 17% at the end of the study (10% in Cit-B12 group, 1 mild, 1 moderate; 30% in placebo group: 3 mild, 0 moderate, Fig. 2). The change occurring between the beginning and the end of the study was statistically significant for Cit-B12 group (*P* = 0.05).

Throughout the study period, a significantly reduction of conjunctival staining was also observed in Cit-B12 group (*P* = 0.04).

Comparing patients with normal and abnormal OSDI at baseline, we found out an association between abnormal OSDI and esthesiometry. Cornea sensitivity was 21 ± 12 mm in patients with abnormal OSDI, and 39 ± 18 mm in those with normal OSDI (*p* = 0.003). The difference in cornea sensitivity between these two groups was still present at the second visit (at month 4, 28 ± 11 mm in abnormal OSDI; 48 ± 14 in normal OSDI, *p* = 0.0001; at month 8, 45 ± 9 in abnormal OSDI; 51 ± 11 in normal OSDI, *p* = 0.08). No other significant differences were found in the study.

Both study treatments had high tolerability, as no patients discontinued the study due to side effects; a mean of 9 ± 15 administrations were missed between 2 consecutive visits, with no statistically significant differences in the two groups (*P* > 0.60).

## Discussion

This is the first paper to test the effects of topical citicoline and vitamin B12 as neuroregenerative agents for the ocular surface, using diabetes as a model of axonal damage. Our results support this neuroregenerative action, as we showed the superiority of Cit-B12 vs placebo in restoring both corneal nerve morphology and function.

In the group receiving topical citicoline and vitamin B12 over a 18-month period, a progressive improvement of axonal density and a corresponding amelioration of corneal sensitivity were shown. At the beginning of the study, Cit-B12 group had low nerve densities and sensitivities (although the study was designed not to have statistically significant SBP differences); at the end of follow up, these parameters were overlapping in the two groups. Percentage improvement of nerve density at the end of the study vs baseline was significantly higher with Cit-B12 than placebo (33% vs 15% respectively). This corresponded to an amelioration of corneal sensitivity of + 150% for Cit-B12 compared with + 44% for placebo. Of note, in the Cit-B12 group mean sensitivity shifted from 28 ± 18 mm at the beginning of the study (corresponding to corneal hyposensitivity) to 52 ± 10 mm (ie normal sensitivity) at the end of the study.

Citicoline may promote corneal nerve repair and axonal regrowth being an essential precursor in the synthesis of phosphatidylcholine (a component of cell membranes), cardiolipin, and sphingomyelin [22, 23]. Also, the intake of vitamin B12 would reduce the levels of oxidative stress occurring in neurodegenerative processes [24]. Some effects on nerve density and corneal sensitivity (although not statistically significant) were also shown on placebo group (0.3% hypromellose, a typical treatment for dry eye disease, DED, in many settings). It is impossible to have a “true” placebo on studies on DED as any eyedrop (also saline solution alone) modifies the ocular surface increasing tear volume and diluting inflammatory molecules. This, in the long term, may also ameliorate ocular surface homeostasis and potentially have a beneficial effect on nerve morphology and function. The short-term beneficial effects of both hypromellose and hyaluronic acid has been previously shown in DED patients [25] but long-term data on structural changes on the ocular surface are currently unavailable.

It is also possible that high variability of confocal data may have played a role in our study; yet the concomitant increase of both morphology and function is more likely a descriptor of a real neuroregenerative effect and not of an artifact.

The changes in ocular surface (both signs and symptoms) were secondary outcomes of the study, and the population was not selected on the basis of these findings. When inspecting mean data no significant changes on ocular surface were shown during the study (Table 5). Yet, studying OSDI questionnaire, we showed that both treatments are useful to ameliorate the symptoms related to dry eye. In fact, according to OSDI classification, the prevalence of dry eye reduced from 36% at baseline to 17% at the end of the study. The change occurring on Cit-B12 group (from 40 to 10%, Fig. 2) was statistically significant. Throughout the study period, Cit-B12 also significantly reduced conjunctival staining.

These results are in agreement with a large part of literature, bringing evidence that nerve trophism plays a key role on the ocular surface homeostasis, and loss of neural network integrity or correct function is a frequent cause of dry eye disease [26], because of reduced corneal sensitivity and lacrimal gland dysfunction with reduced tear production and tear stability [27]. Our population was not selected on the basis of OS findings, and we found out that at baseline patients with abnormal OSDI had significantly lower sensitivity (21 ± 12 mm) than those with normal OSDI (39 ± 18 mm). These results are in contrast with Lyu et al. [28], who suggested that patients with longstanding diabetes may be less symptomatic due to corneal denervation. Another recent paper by Ferdousi et al. [27] found out that DED prevalence in 42 patients with type-1 diabetes was not related with corneal nerve structure and density. Probably, these controversial findings reflect the well-known different effects due to type of diabetes [29], populations (DED on patients with diabetes ranges from 17.5% [30] up to 76.5% [31]), and stage of the disease [32]. Confocal microscopy is a fascinating technique as it allows the in-vivo analysis of the ocular surface at cellular level. Still, several possible limitations may affect these studies: reproducibility may be low, as it is nearly impossible to evaluate exactly the same area of 400 × 400 μm at retest; also, nerve tracing is subjective and may be poorly reproducible. In this paper, we tried to protect our observations including 9 cornea fields at each visit - a fact that has been shown to reduce test-retest variability [21]. Also, we traced the fibers using NeuronJ software, which automatically measures nerve length. Previous to the study, we tested variability of confocal microscopy in two consecutive sessions, and also both inter- and intra-evaluators reading agreement with ImageJ; overall agreement ranged from good to excellent. In spite of our attempts to increase the validity of study measurements, confocal data had high variability; still the association between morphological and functional amelioration seems to corroborate the quality of our dataset despite confocal variability.

Another possible limitation of the study was the difference (though not statistically significant) of nerve density at the beginning of the study in the two groups. This difference was due to the necessity of balancing at baseline two other features which we considered as critical in the study (duration of the disease and number of cases of insulin-dependent diabetes). Weighted randomization on such a small population could not prevent the presence of a clinically relevant difference of nerve density at baseline. Such a difference may have supported a higher chance of amelioration in the group with lower baseline (Cit-B12). Yet, it should be noted that also esthesiometry data support the superiority of active treatment over placebo.

Finally, this pilot study evaluated just a subgroup of diabetic patients. These patients had received retinal laser photocoagulation, which has been found to be associated with conspicuous corneal nerve damage [27]; in fact FLD at baseline was two times lower than normal [33]. The results of this subgroup of patients is hardly generalizable to the whole population of patients with diabetes. Also, our analysis combined both type I and II diabetes; even though we found no differences at confocal microscopy between the two types of diabetes (data not shown), the pathogenesis of neuropathy and the effects of treatments may be very different in the two populations.

Future studies will need to confirm our findings and evaluate the possible efficacy of citicoline and vitamin B in different populations (low or absent nerve damage; moderate - severe DED; patients with neurotrophic corneal ulcers). Future investigations may also explore other corneal features (ie the presence of neuromas), which were not considered in this study and may help in clarifying the course of diabetic neuropathy and the effects of potential treatments.

## Conclusions

This pilot study suggests for the first time a positive effect of topical citicoline and vitamin B12 in ameliorating both corneal nerve density and sensitivity in patients with diabetes; such changes ameliorated the homeostasis of the ocular surface. Our findings raise a number of possible fields of study for topical citicoline, such as the effects on other conditions on which corneal nerves are acutely affected (refractive and corneal surgery) or infected (herpes infections), and the utility on patients complaining for eye pain or severe dry eye with abnormal nerve regulation.

## Data Availability

The datasets during and/or analysed during the current study available from the corresponding author on reasonable request.

## Abbreviations

Cit-B12: Topical 2% citicoline, 0.2% hyaluronic acid and 0.02% cyanocobalamin
DED: Dry eye disease
FLD: Fiber length density
OSDI: Ocular Surface Disease Index questionnaire
SBP: Corneal sub-basal plexus
TBUT: Tear film fluorescein break-up time
